# Serologic response to VDRL in infants with congenital syphilis: ceftriaxone vs. penicillin

**DOI:** 10.1016/j.jped.2026.101530

**Published:** 2026-04-07

**Authors:** Ana Nery Melo Cavalcante, Maria Alix Leite Araújo, Beatriz Sobreira Camilo Soares, Marina Arrais Nobre, Rosa Lívia Freitas de Almeida

**Affiliations:** a Universidade de Fortaleza, Fortaleza, CE, Brazil; b Sociedade Brazileira de Pediatria (SBP), Fortaleza, CE, Brazil; c Universidade de Fortaleza, Postgraduate Programme, Fortaleza, CE, Brazil; d Tulane University, New Orleans, USA; e Universidade Federal do Ceará, Fortaleza, CE, Brazil

**Keywords:** Congenital syphilis, Ceftriaxone, VDRL, Infectious diseases, Penicillin

## Abstract

**Objective:**

Penicillin remains the only safe and effective drug recommended for treating syphilis in pregnant women and congenital syphilis (CS). Nevertheless, alternative therapies are needed when penicillin is contraindicated, such as in allergic reactions, or unavailable due to supply limitations. This study aimed to compare the serological response to the Venereal Disease Research Laboratory (VDRL) test in infants reported with CS and treated with either ceftriaxone or penicillin.

**Method:**

A non-competitive cohort study was conducted in three public maternity hospitals in Fortaleza, Ceará, Northeast Brazil. Data were extracted from notification forms in the Notifiable Diseases Information System (SINAN), medical records from maternity hospitals and outpatient clinics, and electronic records from primary care units. The serological response of infants treated with ceftriaxone and penicillin was assessed. An adequate response was defined as two consecutive negative VDRL tests performed at the intervals recommended by the Brazilian Ministry of Health. Survival curves were estimated using the Kaplan-Meier method.

**Results:**

Among 383 infants reported with CS, 56 (14.6 %) underwent VDRL testing in accordance with Ministry of Health guidelines. Of these, 19 (33.9 %) received ceftriaxone and 37 (66.1 %) penicillin. No statistically significant difference in time to VDRL negativity was observed between the groups (log-rank *p* = 0.73). The median time to negativity was 3 months in both cohorts.

**Conclusions:**

Serological response to VDRL was comparable between infants treated with ceftriaxone and those treated with penicillin. No cases of kernicterus or other CS-related complications occurred among ceftriaxone-treated infants.

## Introduction

Congenital syphilis (CS) is the result of vertical transmission of *Treponema pallidum* from the mother to the fetus during pregnancy. If untreated or inadequately treated, pregnant women with syphilis have a 52 % risk of adverse outcomes in pregnancy and childbirth, including miscarriage, stillbirth, neonatal death, prematurity, low birth weight and other clinical symptoms [1,2].

Penicillin is the drug of choice for treating all forms of syphilis and should be used in pregnant women and infants [3]. However, its use is limited in cases of allergic reactions to the drug [4] and in the context of shortages, such as those faced by several countries, including Brazil, during the period of 2014 to 2016 [5,6]. Countries facing penicillin shortages have used ceftriaxone, amoxicillin and erythromycin [6] as alternative drugs to treat syphilis in pregnant women, despite being unaware of their efficacy and safety, particularly in preventing CS.

Ceftriaxone is a drug with good central nervous system (CNS) penetration, a long half-life (it can be given once a day), [4] and has been used as an alternative to penicillin in the treatment of acquired syphilis and neurosyphilis [4]. However, its cost is higher and its efficacy in the treatment of syphilis in pregnant women and CS remains controversial [7]. Limited published data, primarily in the form of case reports, describe the use of ceftriaxone for treatment of maternal syphilis and CS [[8], [9], [10], [11], [12], [13]].

In Brazil, the Ministry of Health (MoH) recommended prioritizing the use of benzathine penicillin in pregnant women with syphilis during the penicillin shortage period, and ceftriaxone as an alternative treatment for newborns (NB) with CS when crystalline penicillin G and procaine penicillin G were unavailable [14]. It also recommended strict clinical and laboratory follow-up of infants treated with ceftriaxone, given the lack of scientific evidence of its efficacy in treating CS [15].

The use of ceftriaxone in the neonatal period may lead to toxicity, especially in premature infants, due to the possibility of bilirubin displacement at albumin binding sites, leading to the development of kernicterus (bilirubin impregnation of the basal ganglia of the brain). In addition, coadministration with calcium-containing solutions in neonates may result in the formation of insoluble ceftriaxone calcium compounds [16,17].

Given the lack of scientific evidence, the aim of this study is to compare the serological response, measured by titration of the VDRL test, in infants with reported CS treated with ceftriaxone and those treated with penicillin, in order to fill gaps and contribute to scientific knowledge that can guide the treatment of CS in the context of penicillin shortage.

## Material and methods

This is a non-competitive cohort study conducted in three public secondary care maternity hospitals in the municipality of Fortaleza, Ceará, in the northeast of Brazil. These facilities are responsible for about 25 % of all births and report around 23.8 % of CS cases in the municipality. They provide specialized outpatient follow-up through clinical and laboratory evaluation and treatment of infants whose mothers were diagnosed with syphilis during pregnancy.

Data were collected between January and July 2018. First, infants with CS were identified through the notification forms of the Notifiable Diseases Information System (SINAN). Then, data on the performance and results of VDRL tests in newborns diagnosed with CS were collected from the inpatient birth medical records of the maternity hospitals, and complemented with the electronic medical records of the primary care center of the Municipal Health Service (SMS), where infants received outpatient pediatric follow-up care. The definition of CS cases was based on the clinical and epidemiological assessment of the mother (untreated or inadequately treated during pregnancy), the physical examination and the results of laboratory and radiological tests of the infants [18].

The analysis considered two groups: infants treated exclusively with ceftriaxone at birth between May 2015 and December 2016, when penicillin was in short supply (ceftriaxone group); and infants treated with crystalline or procaine penicillin at birth between September 2013 and April 2015, before the shortage (penicillin group). The reason for including infants in the penicillin group in the period before the shortage was that there was an overall shortage of this drug in the maternity hospitals studied in 2015 and 2016. Infants who died and those who did not have two consecutive VDRL tests were excluded.

The 2015 guideline [14] recommended ceftriaxone at 25–50 mg/kg per dose, administered every 24 h intravenously or intramuscularly for 10–14 days, without distinction for neurosyphilis. In 2016 [15], a revised regimen was established: 75 mg/kg/day every 24 h for 10–14 days, while neurosyphilis required 100 mg/kg on the first day, followed by 80 mg/kg every 24 h, administered intravenously for the same duration. The dosage of crystalline penicillin remained unchanged across both guidelines: 50,000 IU/kg every 12 h during the first seven days of life and every eight hours thereafter, for 10 days, intravenously, including in neurosyphilis. Procaine penicillin was prescribed at 50,000 IU/kg per dose every 24 h for 10 days intramuscularly and was likewise indicated for neurosyphilis.

Outpatient follow-up of the infants was analyzed in relation to VDRL tests at 1, 3, 6, 12 and 18 months, presence of signs and symptoms of CS, occurrence of kernicterus, need for re-treatment of CS, and age at discharge due to an adequate serologic response (seroreversion). Infants with two consecutive negative VDRL tests were considered to have an adequate serological response, and VDRL monitoring was subsequently discontinued [15]. Infants who were born with a negative VDRL result were included in the analysis due to the possibility of the test becoming positive at a later date [19]. Therapeutic failure was defined as a rising VDRL titer or the absence of seroreversion by 18 months of age [18].

The birth conditions of the babies were also analyzed, and the variables considered were: weight classification according to gestational age (Small for Gestational Age- SGA, Appropriate for Gestational Age- AGA, Large for Gestational Age- LGA) weight, gestational age, Venereal Disease Research Laboratory (VDRL) test titre, presence of CS signs and symptoms, blood count, cerebrospinal fluid (CSF) and long-bone X-ray results.

Infants were considered symptomatic if they had at least one of the following signs and symptoms: low birth weight, small for gestational age, pneumonia alba, prematurity, hepatomegaly with or without splenomegaly, characteristic skin changes (such as palmoplantar pemphigus), serosanguineous rhinitis, or direct or indirect hyperbilirubinemia (at phototherapy threshold). Blood count changes were: anemia, thrombocytopenia, leukocytosis or leukopenia. Neurosyphilis was considered if the CSF met any of the following criteria: protein > 150 mg/dL, white blood cell count > 25 cells/mm³, or a reactive VDRL. Long bone radiographs were considered abnormal if osteitis, periostitis, or osteochondritis were present [20].

The Statistical Package for Social Sciences (SPSS) software, version 25.0, was utilized for data analysis. Numerical data were expressed as mean and standard deviation (SD) and, in the absence of a normal distribution, as median and 25th-75th interquartile range. Fisher's exact test was used for comparative analysis between categorical variables and types of treatment. For all tests, an alpha of <0.05 was considered necessary to reject the null hypothesis. Survival curves were plotted using the Kaplan-Meier method, and comparisons between treatment groups were made using the log-rank test, with two consecutive negative tests considered the outcome. Additionally, Cox proportional hazards models incorporating treatment group and low birthweight were fitted to estimate adjusted hazard ratios for time to VDRL negativity.

This study was approved by the Research Ethics Committee of the University of Fortaleza (UNIFOR), opinion no 2.505.247, and conducted in accordance with Resolution 466/2012 of the National Health Council. All data were anonymized and stored securely to ensure confidentiality and privacy. The study received financial support from the University of Fortaleza (UNIFOR).

## Results

**Figure 1:**
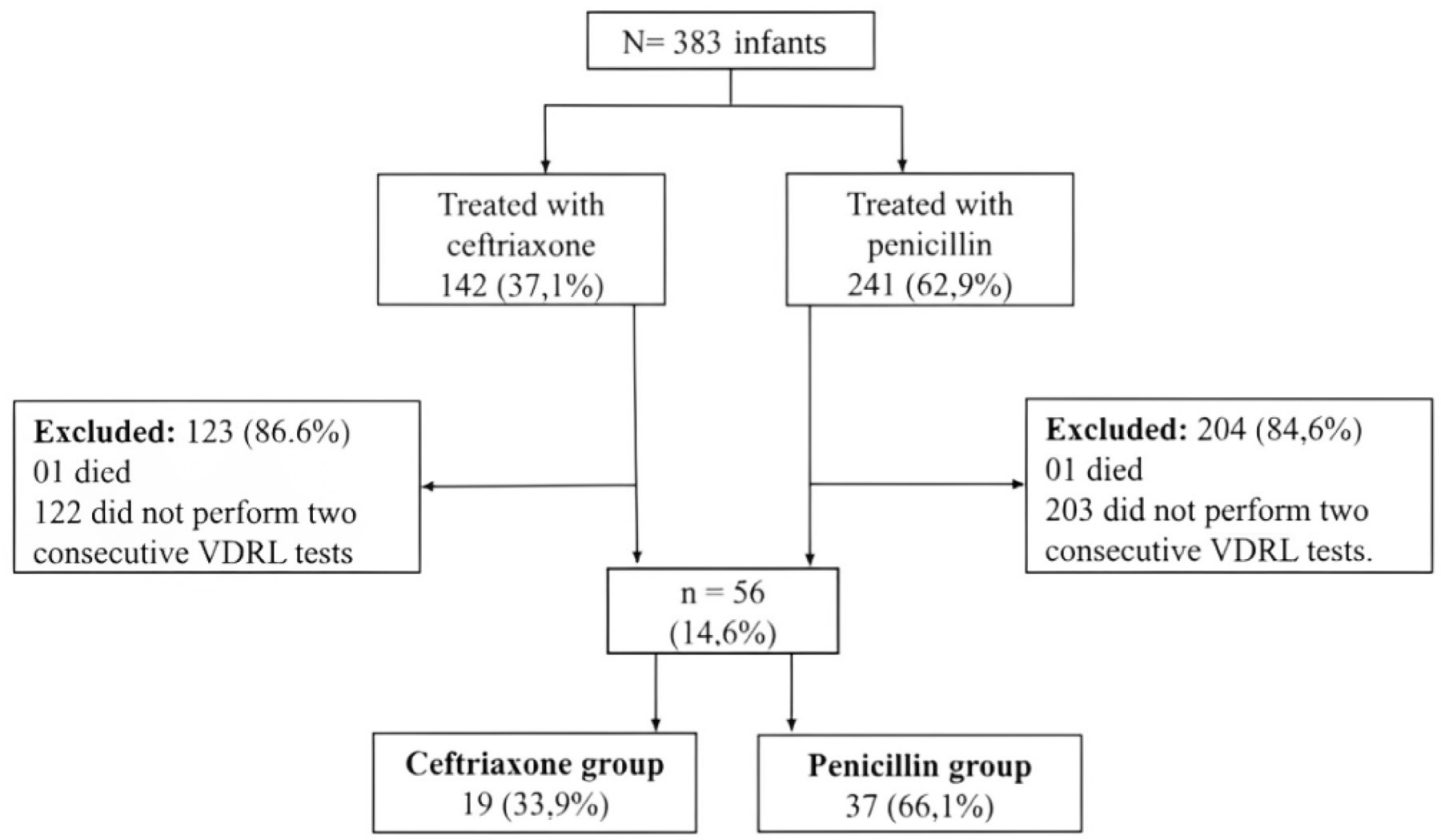
Flowchart of the groups of infants with CS treated with ceftriaxone and infants with CS treated with penicillin according to the results of two consecutive negative VDRL tests. Fortaleza, Ceará, Brazil. 2013–2016.

During the study period, 383 infants were reported and treated for CS. A total of 56 (14.6 %) underwent follow-up VDRL testing as recommended by the MoH. Of these, 19 (33.9 %) were treated with ceftriaxone and 37 (66.1 %) with penicillin. A total of 327 (85.3 %) infants were excluded (Figure 1).

Table 1 shows a bivariate analysis of the birth characteristics of the 56 neonates. The mean weight was 3250 *g* (±563; minimum 1780 and maximum 5160). Five (8.9 %) were classified as small for gestational age (SGA), five (8.9 %) were born underweight (< 2500 g), and six (10.7 %) were born at <37 weeks of gestational age.

**Table 1:** Bivariate analysis of weight classification and gestational age, gestational age, VDRL at birth, clinical signs and symptoms, blood count, cerebrospinal fluid, and long bone radiographs of infants with CS treated with ceftriaxone or penicillin. Fortaleza, Ceará, 2013–2016 (*n* = 56).

		Ceftriaxone Group	Penicillin Group	p
	n/ %	n/ %	n/ %	
**Weight/gestational age classification**				0041
AGA	44/78,6	14/31,8	30/68,2	
SGA	05/8,9	04/80,0	01/20,0	
LGA	07/12,5	01/14,3	06/85,7	
**Birth weight**				0045
< 2500 *g*	05/8,9	04/80,0	01/20,0	
≥ 2500 *g*	51/91,7	15/29,4	36/70,6	
**Gestational age**				0974
Preterm	06/10,7	02/33,3	04/66,7	
Term	50/89,3	17/34,0	33/66,0	
**VDRL at birth**				0780
Non-reactive	22/39,3	08/36,4	14/63,6	
Reactive	34/60,7	11/32,4	23/67,6	
**Presence of signs and symptoms**				0518
No	42/75,0	13/31,0	29/69,0	
Yes	14/25,0	06/42,9	08/57,1	
**Blood count**				0197
Normal	38/73,1	11/28,9	27/71,1	
Altered	14/26,9	07/50,0	07/50,0	
**CSF cytology and/or proteins**				
Normal	33/58,9	14/42,4	19/57,6	0158
Altered	02/3,6	01/50,0	01/50,0	
Not performed	21/37,5	04/19,0	17/81,0	
**VDRL in CSF**				
Non-reactive	35/62,5	15/42,9	20/57,1	0086
Not performed	21/37,5	04/19,0	17/81,0	
**Long Bone Radiograph**				
Normal	52/92,9	18/34,6	34/65,4	0209
Altered	01/1,8	01/100,0	0/0	
Not performed	03/5,4	0/0	03/100,0	

Comparisons between treatment groups were performed using Fisher’s exact test; p-values are exploratory and not adjusted for multiple testing.

AGA, Appropriate for Gestational Age; SGA, Small for Gestational Age; LGA, Large for Gestational Age; CSF, Cerebrospinal fluid.

At birth, 34 infants (60.7 %) had a reactive VDRL result, and 14 (25.5 %) presented with at least one sign or symptom of congenital syphilis (42.9 % in the ceftriaxone group and 57.1 % in the penicillin group). The most frequent findings were jaundice meeting phototherapy thresholds (5/8.9 %) and abnormal blood counts (14/26.9 %). Regarding CSF, cytology or protein was altered in two (3.6 %), not performed in 21 (37.5 %), and VDRL was not performed in 21 (37.5 %). Long bone radiographs were altered in one (1.8 %) and not taken in three (5.4 %).

In analyses stratified by VDRL status at birth (reactive vs. non-reactive), all infants in each stratum and treatment group achieved an adequate serologic response. Among those with a reactive VDRL, 23/23 (100 %) in the penicillin group and 11/11 (100 %) in the ceftriaxone group attained two consecutive non-reactive results by 12 months. Among infants with a non-reactive VDRL at birth, 14/14 (100 %) in the penicillin group and 8/8 (100 %) in the ceftriaxone group likewise demonstrated an adequate response (Supplementary Table S1).

Exploratory analyses stratified by the presence of signs and symptoms at birth (symptomatic vs. asymptomatic, as defined in the Methods) showed an adequate serologic response in all strata and treatment groups. (Supplementary Table S2).

Infants classified as SGA (*p* = 0.041) and those with low birth weight (*p* = 0.044) were more likely to be treated with ceftriaxone at birth. In accordance with the authors’ definition of symptomatic infants in the Methods, low birth weight and SGA are classified as clinical or laboratory signs. In Table 1, however, “presence of signs and symptoms” is a composite variable, whereas birth weight and weight-for-gestational-age categories are reported separately. Consequently, statistically significant differences in low birth weight and SGA may be observed between groups despite no difference in the overall composite “presence of signs and symptoms.”

**Figure 2:**
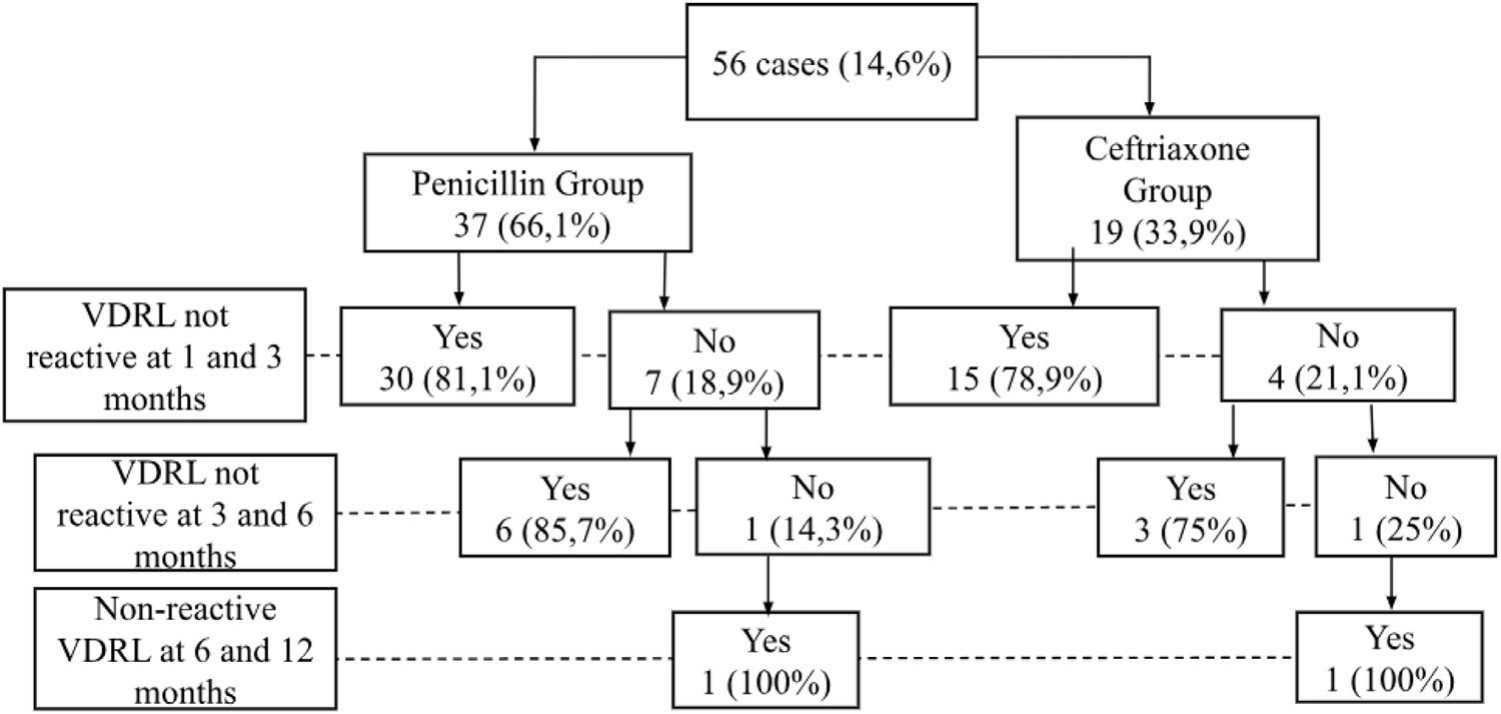
Flowchart of the evolution of VDRL test results in infants in the ceftriaxone and penicillin groups until the age of seroreversion. Fortaleza, Ceará, Brazil. 2013–2016.

At one and three months of age, 30 (81.1 %) and 15 (78.9 %) infants in the ceftriaxone and penicillin groups, respectively, had two consecutive negative VDRL tests. All infants achieved an adequate serologic response (seroreversion) by 12 months (Figure 2).

**Figure 3:**
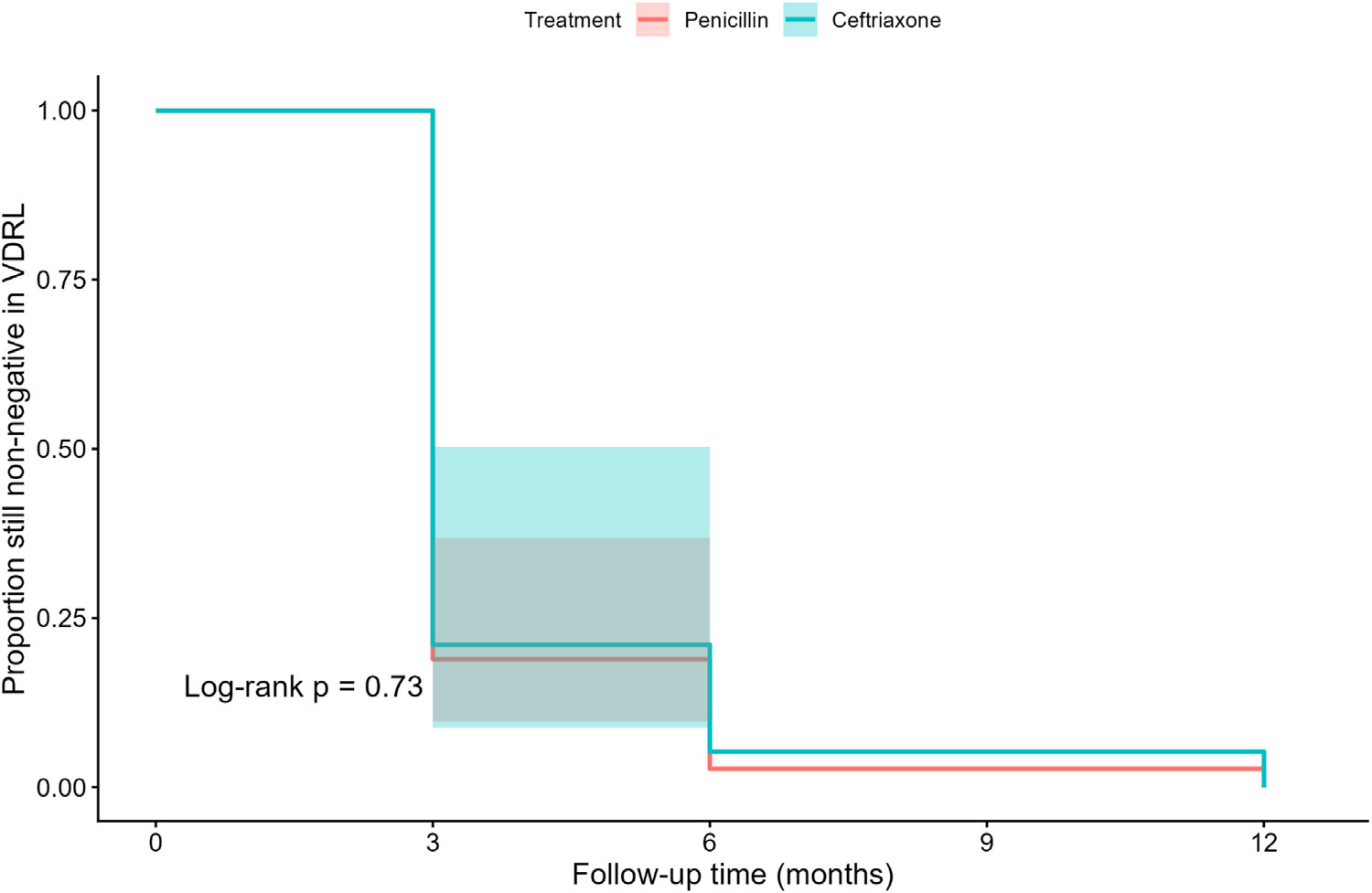
Kaplan–Meier curves for time to VDRL negativity in infants with congenital syphilis treated with ceftriaxone or penicillin (log-rank *p* = 0.73). Fortaleza, Ceará, Brazil, 2013–2016 (*n* = 56).

In the Kaplan–Meier analysis, the median time to VDRL seronegativity (seroreversion) was 3 months (95 % CI, 3–3) in the penicillin group and 3 months (95 % CI, 3–6) in the ceftriaxone group, with no statistically significant difference between survival curves (log-rank *p* = 0.73; Figure 3). In the Cox proportional hazards model including treatment group and low birth weight, ceftriaxone was not associated with a different time to seroreversion compared with penicillin (adjusted hazard ratio [HR], 0.84; 95 % CI, 0.47–1.51; *p* = 0.57), nor was low birth weight significantly associated with time to seroreversion (adjusted HR, 1.88; 95 % CI, 0.71–4.99; *p* = 0.21; Table 2). No cases of kernicterus, sequelae, or retreatment were observed among infants treated with ceftriaxone.

**Table 2:** Cox proportional hazards model for time to VDRL negativity among infants with congenital syphilis treated with ceftriaxone or penicillin. Fortaleza, Ceará, Brazil, 2013–2016 (*n* = 56).

Variable	Adjusted hazard ratio (95 % CI)	p-value
Ceftriaxone vs. penicillin	0.84 (0.47–1.51)	0.57
Low birthweight (yes vs. no)	1.88 (0.71–4.99)	0.21

## Discussion

In this retrospective review of a small convenience sample of infants with congenital syphilis, no statistically significant differences were observed in serologic response or time to VDRL seronegativity between those treated with ceftriaxone and those treated with penicillin. No cases of kernicterus were reported among infants treated with ceftriaxone.

Given that no studies were identified in the scientific literature that compared the evolution of VDRL titration in infants with CS treated with ceftriaxone and penicillin, this research brings important results by providing a comparison of the serologic response to the VDRL test in infants treated with these drugs. It also draws attention to the importance of developing prospective clinical trials to evaluate the efficacy of this drug in the treatment of CS, considering the need to identify another alternative drug, due to the possibility of new episodes of penicillin shortages.

Currently, ceftriaxone has no proven efficacy in the treatment of syphilis in pregnant women and CS, and its use is recommended only when benzathine, crystalline, and procaine penicillin G are unavailable [20]. Regarding acquired syphilis, studies show that its efficacy is similar to that of penicillin [4,20] and the Brazilian MoH [18] makes it an alternative drug for treating cases of neurosyphilis and in cases of allergic reaction to penicillin, a situation that can occur in 10 % of patients [4,21]. For its use in pregnant women with syphilis, a case study conducted in Tokyo, Japan, showed that the conceptus of a mother treated with ceftriaxone showed no symptoms of CS, did not require treatment, and the non-treponemal test was negative at four months [8].

Brazil's penicillin shortage problem began in May 2014, and the Health Ministry only issued its first regulation in October 2015 [14]. Research carried out in the city of Fortaleza, in northeastern Brazil, showed that during this period, different drugs and therapeutic regimens were used to treat infants with CS [13] and that the choice of therapeutic regimen was left to the discretion of neonatologists, as well as the drugs available in maternity hospitals.

Considering that two consecutive negative VDRL tests are one of the criteria for infants to be considered with an adequate serologic response [18], among this small sample of infants with congenital syphilis and adequate post-treatment VDRL follow-up, 100 % of infants treated with either penicillin or ceftriaxone achieved an adequate serological response and were considered seroreversed. In terms of evaluating negative VDRL tests in infants with CS treated with penicillin, a study conducted in Porto Alegre, Rio Grande do Sul, evaluated the follow-up and reversal of VDRL in 119 infants. It demonstrated that in 81.5 % of cases, this test was negative at three months of the child's life [22].

It is noteworthy that only 14.6 % of infants reported and treated for congenital syphilis had adequate follow-up and were discharged as seroreversed, defined by two consecutive negative VDRL tests. Previous studies indicate that adherence to outpatient follow-up for infants with congenital syphilis is low [23,24] or inadequate even among those treated with penicillin [25]. The predominance of asymptomatic newborns [26] may contribute to families underestimating the importance of follow-up. It is essential to identify strategies to improve communication with this population, enhance adherence to scheduled medical visits, and thereby minimize losses [27].

Infants should be properly monitored after discharge from the hospital, even if they are negative for VDRL and asymptomatic at birth [28]. to exclude the diagnosis of CS. A study conducted in Montes Claros, Minas Gerais, southeastern Brazil, found that 45.4 % of infants with CS did not have blood counts, long bone radiographs, and cerebrospinal fluid tests [29]. In the case of infants with neurosyphilis, the Brazilian MoH guidelines recommend that CSF should be repeated every six months until the CSF normalizes during the follow-up period [12].

During prenatal care, pregnant women diagnosed with syphilis should be counseled regarding the importance of postnatal monitoring of the infant. The failure of health services to ensure such follow-up is particularly concerning, as it reflects noncompliance with Ministry of Health recommendations [15,18]. This concern is further aggravated in the present cohort, given that some infants were treated with ceftriaxone, a drug for which no robust scientific evidence supports its use in the treatment of congenital syphilis.

Investments should be made in the follow-up of infants with CS, particularly to ensure that appointments are made when they are discharged from the maternity hospital. It is necessary to improve the communication process between maternity wards and primary care, and to train the professionals who carry out the follow-up.

With respect to the statistically significant differences in birth weight and weight-for-gestational-age classification, infants treated with ceftriaxone more frequently exhibited low birth weight and were small for gestational age. As defined in the Methods, these conditions are included among the clinical and laboratory signs used to classify infants as symptomatic; however, in Table 1, “presence of signs and symptoms” is a composite variable, whereas birth weight and weight-for-gestational-age classification are presented separately, explaining why differences are observed for the individual variables but not for the composite measure. Given the small sample size and sparse cells across several baseline variables, these findings should be interpreted cautiously as exploratory. For this reason, the authors did not fit a multivariable model including all baseline characteristics, as it would lack statistical robustness and could yield misleading results.

A limitation of this study is the exclusion of infants treated with ceftriaxone who lacked adequate follow-up, which may have biased the results. Exposure was determined by the period of national penicillin shortage; thus, infants in the ceftriaxone group were born later in the study period (2015–2016), whereas those in the penicillin group were born earlier (2013–2015). Treatment group and calendar period were intrinsically linked and could not be modeled independently. Consequently, unmeasured temporal or site-related changes in clinical practice, case mix, or follow-up may have influenced outcomes, and residual confounding cannot be excluded. These infants require further evaluation due to the risk of late manifestations and irreversible sequelae such as deafness, intellectual disability, seizures, and bone and ophthalmological abnormalities [26]. Therefore, it is necessary to assess the health status of all infants treated with ceftriaxone during the period of penicillin shortage.

Time to VDRL seronegativity was similar between ceftriaxone- and penicillin-treated infants, and no cases of kernicterus or sequelae were observed in the ceftriaxone group. As this was a retrospective study, definitive conclusions regarding ceftriaxone efficacy for congenital syphilis cannot be drawn; prospective trials are required. Nevertheless, ceftriaxone may represent a therapeutic alternative during periods of penicillin shortage.

## Authors’ contributions

Ana Nery Melo Cavalcante: Study design and conception, data acquisition, analysis and interpretation, article writing, critical review of relevant intellectual content, final approval of the version to be submitted.

Maria Alix Leite Araújo: Data analysis and interpretation, critical review of relevant intellectual content, final approval of the version to be submitted.

Beatriz Sobreira Camilo Soares: Data acquisition, data analysis and interpretation, article writing, critical review of relevant intellectual content, final approval of the version to be submitted.

Marina Arrais Nobre: Study conception and design; data acquisition; data analysis and interpretation; article writing; critical review of relevant intellectual content; final approval of the version to be submitted.

Rosa Lívia Freitas de Almeida: Study design and outline, data analysis and interpretation, critical review of relevant intellectual content, final approval of the version to be submitted.

## Funding

This study was financially supported by the University of Fortaleza (UNIFOR), a not-for-profit institution.

## Data availability statement

The data that support the findings of this study are available from the corresponding author.

## Conflicts of interest

The authors declare no conflicts of interest.

## Appendix Supplementary materials

Image, application 1
