# Supplementary material for: Serologic response to VDRL in infants with congenital syphilis: ceftriaxone vs. penicillin
Source: J Pediatr (Rio J). 2026 Apr 7;102(3):101530. doi: 10.1016/j.jped.2026.101530 (PMC13090585; doi:10.1016/j.jped.2026.101530)
Supplement: Supplementary file 1 [file mmc1.docx]

**JPED-D-25-00368_ Supplementary Material**

# **Supplementary Table S1** Baseline VDRL result at birth and serologic response according to treatment group in infants with adequate VDRL follow-up. Fortaleza, Ceará, 2013-2016 (n = 56).

| VDRL at birth | Treatment group | Infants with adequate follow-up, n | Infants with adequate serologic response*, n | Infants with adequate serologic response, % |
| --- | --- | --- | --- | --- |
| Reactive | Penicillin | 23 | 23 | 100 |
| Reactive | Ceftriaxone | 11 | 11 | 100 |
| Non-reactive | Penicillin | 14 | 14 | 100 |
| Non-reactive | Ceftriaxone | 8 | 8 | 100 |

*Adequate serologic response defined as two consecutive non-reactive VDRL tests by 12 months of age.

# **Supplementary Table S2** Clinical status at birth (symptomatic vs asymptomatic) and serologic response according to treatment group in infants with adequate VDRL follow-up (n = 56). Fortaleza, Ceará, 2013-2016 (n = 56).

| Clinical status at birth | Treatment group | Infants with adequate follow-up, n | Infants with adequate serologic response*, n | Infants with adequate serologic response, % |
| --- | --- | --- | --- | --- |
| Symptomatic | Penicillin | 8 | 8 | 100 |
| Symptomatic | Ceftriaxone | 6 | 6 | 100 |
| Asymptomatic | Penicillin | 29 | 29 | 100 |
| Asymptomatic | Ceftriaxone | 13 | 13 | 100 |

*Adequate serologic response is defined as two consecutive non-reactive VDRL tests by 12 months of age.
